# Non-Medical Opioid Use in Ethiopia: An Exploratory Descriptive Study

**DOI:** 10.4314/ejhs.v36i1.4

**Published:** 2026-01

**Authors:** Melaku Tesfaye, Yonas Baheretibeb, Tsimona Dinku, Awoke Mihretu

**Affiliations:** 1 Department of Psychology, College of Social Sciences and Humanity, Mizan-Tepi University, Mizan Teferi, Ethiopia; 2 Department of Psychiatry, College of Health Sciences, Addis Ababa University, Addis Ababa, Ethiopia

**Keywords:** Opioid Use, illicit drug use, Problematic Opioid use, non-medical opioid use, tramadol, Ethiopia

## Abstract

**Background:**

Non-medical opioid use is linked to severe consequences such as addiction, overdose, and infectious diseases, including human immunodeficiency virus (HIV), hepatitis B virus (HBV), and hepatitis C virus (HCV), particularly through injection practices. In Ethiopia, limited evidence exists on non-medical opioid use, and harm-reduction programs are absent. This study aimed to explore the experiences of non-medical opioid users seeking healthcare in Addis Ababa, Ethiopia.

**Methods:**

A phenomenological qualitative study design was employed, complemented by descriptive quantitative data. We conducted qualitative in-depth interviews with sixteen purposively selected participants and reviewed hospital data (n = 47). Thematic analysis was used for the qualitative data, while descriptive statistics summarized the quantitative findings.

**Results:**

Five themes emerged from the qualitative analysis: patterns of opioid use, reasons for use, strategies for accessing opioids, perceived consequences, and help-seeking behaviours. Quantitative data showed that 47 opioid users received healthcare services over the past three years in hospitals in Addis Ababa. Forty-three percent of these individuals were healthcare professionals. Sixty-six percent used one type of opioid, predominantly tramadol (18), while 24 of the 47 reported polysubstance use. Daily opioid use was common, reported by 76.6% of participants.

**Conclusions:**

Non-medical opioid use poses significant life-threatening risks and multidimensional challenges, necessitating immediate intervention. Further large-scale studies on the burden of opioid use, as well as intervention research aimed at improving care and developing harm-reduction guidelines, are essential.

## Introduction

Non-medical opioid use is defined as taking someone else's prescription medication or taking medication solely for the experience it produces (1). Non-medical opioid use can lead to opioid use disorder, opioid dependence, and other health problems (2). Opioid use disorder is characterized by a loss of control over use and significant impairment in social, occupational, or other important areas of functioning (3). According to the United Nations Office on Drugs and Crime (UNODC) 2020 global drug report, sixty-one million individuals worldwide use opioid drugs (4). This report further indicates that the estimated prevalence of opioid use disorder is 0.29% (5).

According to World Health Organization (WHO) estimates, in 2019 opioids were responsible for 80% of the 600,000 drug-related deaths worldwide, with approximately 25% of these deaths resulting from opioid overdose (2). Opioid use disorders account for 40% of the healthy years of life lost due to drug use. In 2021, of the 27.7 million disability-adjusted life years (DALYs) attributable to drug use, 11.2 million were linked to opioid use disorders. In addition, approximately 1.6 million healthy years of life were lost to cancers, mainly liver cancer resulting from hepatitis C, and these losses were primarily associated with injecting drug use (6).

Problematic opioid use among adults with chronic non-cancer pain has been estimated at 36.3% (7). Furthermore, high-income countries account for a disproportionate share of global opioid consumption, while low-income countries represent only 6% of worldwide opioid use (8, 9).

There is evidence that the prevalence of problematic opioid use—defined as a pattern of opioid use that significantly increases the risk of physical and mental health consequences for the user and others (10, 11)—is increasing across Africa (8), including Ethiopia (12, 13).

Non-medical use of opioids is known to increase the risk of overdose-related death (4, 14, 15). Additionally, the use of injectable opioids poses a substantial risk for the transmission of infectious diseases such as HIV, HBV, and HCV. This risk is amplified by syringe sharing, which may result from limited access to needle and syringe programs (NSPs), legal restrictions on injection drug use, and barriers to treatment for HBV, HCV, and HIV (12, 13, 16, 17).

Illicit and non-medical opioid use are also the leading causes of drug-related deaths globally. The continuing rise in opioid-related mortality, particularly in North America, Australia, and Europe, represents a major public health challenge (14).

Estimates of problematic opioid use have been reported across Africa (8, 17–19), including in Ethiopia (20). A review study indicated that opioid consumption for non-clinical indications has increased in Africa as global opioid trafficking routes have evolved to utilize African trading networks (8). In Ethiopia, a small-scale study reported an increase in the use of injectable opioids and associated physical health consequences, including HIV and other infections such as HBV, HCV, and syphilis (12, 13).

Another study on tramadol abuse in Ethiopia reported that 46 university students (11.4%) out of 402 had used tramadol at least once in their lifetime. Factors significantly associated with tramadol abuse included having friends who use drugs, prior knowledge about tramadol, and a history of tramadol use following a physician's prescription.

Although some studies have estimated the magnitude of non-medical opioid use, a substantial knowledge gap remains in Ethiopia regarding how individuals obtain opioids, their life trajectories with opioid use, subjective suffering, psychosocial consequences, coping strategies, user perceptions, help-seeking behaviours, and the availability of healthcare services. Qualitative studies, such as the present one, are critical for developing a deeper understanding of the lived experiences of individuals who use opioids. Therefore, this study aimed to explore the experiences of individuals engaged in non-medical opioid use, focusing on: (1) patterns of use, (2) motivations for nonprescription use, (3) psychosocial impacts, and (4) treatment-seeking behaviours in selected hospitals in Addis Ababa, Ethiopia.

## Methods

**Study design**: We employed an exploratory descriptive study design using qualitative interviews to explore the experiences of individuals who use opioids. This approach enabled an in-depth examination of participants' lived experiences, the meanings they assign to those experiences, and how they interpret them (21). A descriptive quantitative design was also used to support the qualitative findings.

**Study setting:** The study was conducted in two purposively selected referral hospitals: Zewditu Memorial Hospital (ZMH) and St. Paul's Hospital Millennium Medical College (SPHMMC) in Addis Ababa, the capital city of Ethiopia. Both hospitals have drug and alcohol addiction detoxification centers and receive referrals from primary care facilities. Services are provided on an inpatient basis, with an average duration of approximately 10 days.

**Sampling**: A total of 16 participants were selected for the qualitative component from both settings: 10 from ZMH and 6 from SPHMMC. Participants were selected using a purposive sampling technique based on eligibility criteria. First, participants had a history of opioid use. Second, personal contact information or addresses were available in the medical records to allow tracing, and participants were willing to take part in the study. Participants were recruited from diverse backgrounds in terms of gender, age, occupation, residence, and other characteristics until thematic saturation was achieved. Five participants were traced through contact information, while the remaining participants were recruited during admission for detoxification.

For the quantitative component, we reviewed records of all individuals who sought opioid detoxification services at the two health facilities. Participants in the qualitative and quantitative components were mutually exclusive. Interview sample size was determined based on the principle of theoretical saturation. The threshold for medical chart review was set at three years, as individuals who sought care earlier may now be living under different circumstances, and electronic medical records were more reliably maintained during this period.

**Eligibility criteria**: Participants with experience of non-medical opioid use, who had received healthcare within the past three years, were aged over 18 years, and were able to communicate in Amharic were eligible for inclusion. On the other hand, participants with a diagnosis of severe mental illness who were unable to provide consent and whose diagnoses were recorded alongside other comorbid disorders in the patient registration book were excluded.

**Data collection methods**: In-depth interviews were conducted in Amharic to explore participants' experiences with non-medical opioid use. Interviews were audio-recorded, and field notes were taken. Quantitative descriptive data were obtained through medical chart reviews using a pre-prepared checklist to extract sociodemographic characteristics and patterns of opioid use.

**Data management and analysis**: Qualitative data were transcribed verbatim and translated into English by the principal investigator. Accuracy was continuously reviewed through discussions with co-authors, and translation nuances were addressed collaboratively. Data were managed using OpenCode software version 4.03. Thematic analysis followed the six-step approach described by Braun et al., including familiarization with the data, generation of initial codes, theme development, theme review, theme definition and labeling, and report production (22). Quantitative data were analyzed using descriptive statistics, including frequencies and percentages (23).

**Data quality assurance**: To ensure data quality, interviews were conducted in safe, quiet, and private settings. Selected translated excerpts were cross-checked with participants when feasible. The research process was iterative, with all co-authors contributing to coding and theme development. Initial codes were double-coded by the first and last authors, and once consensus was reached, the first author completed the final coding.

**Researcher positionality**: The research team is based within a psychiatry department in Ethiopia, with the primary investigator being a clinical psychologist. We acknowledge that this context may have influenced the study framing, interactions with participants, and interpretation of findings. Nevertheless, the lead author encouraged participants to share their stories using open-ended questions, minimizing researcher influence as much as possible.

**Ethical considerations**: All procedures contributing to this work complied with the ethical standards of relevant national and institutional committees and with the Helsinki Declaration of 1975, as revised in 2013. Ethical approval was obtained from the Research and Ethics Committee of the Department of Psychiatry, Addis Ababa University (MF/PSY/24/14). Additional approvals were granted by the Addis Ababa Health Bureau (AAHB) and St. Paul's Hospital Millennium Medical College (SPHMMC).

Written informed consent was obtained from all participants prior to interviews, and their right to withdraw at any stage was respected. No significant harm to participants was anticipated. Potential risks, including active suicidal behaviours, were assessed, and safety planning was considered; no serious risks were identified. Participants were initially identified through medical records and contacted using phone numbers, following ethical approval for access to contact information. Prior to participation, individuals received information about the study objectives and an information sheet via email. Data were anonymized to maintain confidentiality. Interviews were conducted independently of hospital staff involved in participants' care, and data collectors were not affiliated with the study settings.

## Results

Over the three-year period from 2021 to 2023, a total of 47 opioid users received treatment in both outpatient and inpatient clinics, with 23 treated at ZMH and 24 at SPHMMC. The sociodemographic characteristics of the participants are presented in Table 1.

**Table 1:** Sociodemographic characteristics of participants

Characteristics	Category	Number	Percent
Sex	Male	40	85.1
	Female	7	14.9
Age	18-25	18	38.3
	26-30	20	42.6
	31 and above	9	19.1
Educational Status	Below grade 12	13	27.7
	Diploma	3	6.4
	Degree	22	46.8
	Master and Specialty certificates in medicine	9	19.1
Marital Status	Single	35	74.5
	Married	11	23.4
	Divorced	1	2.1
Occupation	Not recorded	1	2.1
	Student	7	14.9
	Health Professional	20	42.6
	Unemployed	9	19.1
	Others*	10	21.3
Living resident	Addis Ababa	33	70.2
	Outside of Addis Ababa	14	29.8

* family business, messenger, garage work, teaching

**Socio-demographic characteristics:** The majority of participants (22) were university educated, having attained at least an undergraduate degree, and 20 were healthcare workers.

**Patterns of opioid use**: According to data obtained from patients' medical records, participants who visited both hospitals had used either only one type of opioid (31) or multiple types of opioids (at least two kinds) (16). Among those using a single opioid, tramadol was the most commonly used (18), followed by pethidine (10) (Table 2).

**Table 2:** Types of opioids used by the participants

Type of opioid		Number	Percent
Only one opioid use	Tramadol	18	38.3
	Pethidine	10	21.3
	Oxycodone	1	2.1
	Morphine	1	2.1
	Heroin	1	2.1
	Total	31	66
At least two types of opioid use	Tramadol, Pethidine & Morphine	3	6.4
	Pethidine & Tramadol	5	10.6
	Pethidine, morphine, codeine, tramadol	2	4.3
	Pethidine, heroin	1	2.1
	Morphine, pethidine	2	4.3
	Tramadol, heroin	2	4.3
	Morphine, tramadol, heroin	1	2.1
	Total	16	34

Regarding the route of administration, 20 participants reported injecting opioids. Twenty-two participants used opioids more than once daily, and most individuals with opioid use (31) had been admitted up to two times. Over half of the participants (24) had a history of using other psychoactive substances, with 9 reporting the use of more than three different psychoactive substances. Among these psychoactive substances, a high number of participants co-used opioids with tobacco (19) and khat(14).

The qualitative findings revealed that a total of 16 participants were included in the interviews from both study settings: 10 from ZMH and 6 from SPHMMC. Two participants were female, while the remaining fourteen were male. Eight participants were healthcare professionals.

### The patterns of opioid use

Quantitative indicators of participants' opioid use patterns are presented in Table 3.

**Table 3:** Participants' patterns of opioid use

Variables		Number	Percent
Opioid and other substance use	Khat	14	29.8
	Alcohol	11	23.4
	Tobacco	19	40.4
	Others (Cocaine, cannabis, syrup, clonazepam, ketamine, crystal meth, Hashish)	11	23.4
Duration of Opioid Use	Below a year	4	8.5
	Between 1 to 3 years	23	48.9
	More than 3 years	19	40.4
	No record	1	2.1
Frequency of Opioid use	No record	9	19.1
	More than daily*	22	46.8
	Daily	14	29.8
	3-5 times in a week	2	4.3
Forms of Using Opioid	No record	7	14.9
	Tablet	9	19.1
	Injection	20	42.6
	Both	11	23.4
Received type of treatment	No record	2	4.3
	Detox	25	53.2
	Psychotherapy	2	4.3
	Both	18	38.3
Number of Admission to the selected hospital	Up to two times	31	66.0
	More than two times	14	29.8
	Only have OPD Follow-up	2	4.3
Age of starting opioid	Before 18 years	7	14.9
	Between 18 to 29 years	28	59.6
	After 30 years	7	14.9
	No record	5	10.6
Opioid and other psychoactive substance patterns of use			
Absent of other substance use (sub)		23	48.94
≥ 1 opioid & 1 others sub*		4	8.51
≥ 1 opioid & 2 others sub#		7	14.89
≥ 1 opioid & 3 other sub^		4	8.51
≥ 1 opioid & > 3 other sub++		9	19.15

List of Other substances identified through the chart review:
**Stimulants**: Khat* (1), # (4), ^ (4), ++ (5), Tobacco* (2), # (6), ^ (5), ++ (6), cocaine++ (2), crystal meth++ (2) **Depressant**: Alcohol* (3), ^ (2), ++ (3) shisha^ (1), ++ (3), cannabis^ (1), ++ (5) ketamine ++ (1), clonazepam++ (1)

**Binge opioids use**: The most frequently consumed opioids identified by participants were tramadol, pethidine, morphine, heroin, and codeine. Tramadol and pethidine were highlighted as being more easily accessible and were often used in large doses. The following reports illustrate this pattern:

“I used to take 15 to 20 ampules per day, which amounts to 1500 to 2000 mg.” (34-year-old male health professional).

“I was taking up to seven strips per day. One strip contains ten pills, so it was over three thousand milligrams daily.” (22-year-old male student).

Morphine and heroin were used less frequently, primarily because of their higher cost and limited availability.

One participant remarked as:

“I use morphine occasionally because it's expensive and difficult to get regularly.” (20-year-old college student).

In addition to high dosages, participants reported switching between opioids based on availability and perceived potency, with a preference for pethidine.

**Poly-opioid and psychoactive substance use**: Poly-opioid use, defined as the concurrent use of different opioid formulations, was common among participants. Some individuals also mixed opioids with other psychoactive substances, such as alcohol, cannabis, and diazepam.

One participant noted: “We take codeine syrup, mix it with pills, and drink it with alcohol or other substances.” (Male, 20 years old, college student).

Another participant explained: “There were times we mixed opioids with alcohol or cannabis, particularly tramadol and morphine.” (male, 34 years old, health professional).

A disturbing trend was observed in cases where participants combined pethidine with high doses of diazepam during suicide attempts.

One participant described: “I used pethidine and diazepam to try and kill myself. I took 40 mg of diazepam, compared to the usual 5 mg I'd use.” (28-year-old male health professional).

### Choosing specific settings for opioid use

Participants sought secluded and calm environments for opioid consumption, often renting rooms for this purpose.

“I prefer using opioids in silent places, like bathrooms or rooms I've rented for privacy.” (30-year-old health professional).

Others selected secure locations, such as church compounds or their homes, to avoid detection and to ensure consistent access to a safe space for drug use.

**Relapse experiences**: Relapse was commonly reported among participants who attempted to quit opioid use. Workplace exposure was cited as a major trigger for relapsing, particularly due to the easy availability of opioids in medical settings.

A participant reflected:

“I relapsed because I kept seeing opioids at work. It triggered my cravings, especially during depressive episodes.” (26-year-old female health professional).

The reasons for opioid use were also identified.

**Medical use to alleviate pain**: Several participants initiated opioid use for medical reasons, often without adequate investigation. Participants who were healthcare professionals reported prescribing opioids to themselves or obtaining them for pain relief. A 26-year-old female health professional explained: “I started using pethidine for back pain after tramadol failed to help. It was prescribed by a doctor.” (26-year-old female health professional). However, concerns were raised about inappropriate prescribing practices, particularly the use of opioids for uninvestigated or unexplained pain.

**Experimentation and curiosity**: Curiosity and experimentation were additional drivers of opioid use. Participants, especially healthcare professionals, reported exposure to opioids in their work environments, which facilitated experimentation, i.e. A 30-year-old male health professional shared: “During my internship, I was curious, and my friends injected me with opioids. I always wanted to try new things.” (30-year-old male health professional).

**Social influence**: Peer pressure and social modeling played significant roles, particularly among younger participants, i.e. A 23-year-old male student recounted: “I saw other students having fun with tramadol and decided to try it, thinking it wouldn't hurt me.” (23-year-old male student). Participants recovering from alcohol use disorder also reported exposure to opioids such as morphine and tramadol during rehabilitation, which further contributed to opioid use.

**Recreational use and stress relief**: Several participants used opioids recreationally, seeking pleasure or relief from stress, grief, or dissatisfaction with other substances, i.e. One participant, a 28-year-old health professional, stated: “I tried pethidine for fun after a long hospital shift. We were using khat, alcohol, and cannabis, but it wasn't satisfying, so we decided to try opioids.” (28-year-old health professional).

**Craving and retaining pleasure**: Participants described intense cravings following initial opioid use, which drove continued consumption, i.e. A 30-year-old male health professional recounted: “The euphoria was incredible. I started using more ampules to get that same feeling again.” (30-year-old health professional).

**Perceived benefits of opioid use**: Some participants believed opioids enhanced their productivity, particularly those in creative professions such as music, i.e. A 30-year-old male music producer explained: “Taking opioids helps me rap better. It inspires new ideas when recording.” (30-year-old male music producer). Others noted the ease of accessing opioids through local dealers or healthcare professionals, particularly tramadol and pethidine.

**Fear of withdrawal symptoms**: Participants expressed considerable anxiety about opioid withdrawal, describing symptoms such as respiratory depression, headache, diarrhea, involuntary movements, and extreme discomfort, i.e. One participant, a 34-year-old male health professional, explained: “I had respiratory depression, diarrhea, and involuntary movements. I couldn't breathe properly, and that's why I kept taking pethidine.” (34-year-old male health professional). Psychological difficulties, including depression and loss of social confidence, were also reported during attempts to discontinue opioid use.

**Strategies for accessing opioids**: Participants described a range of strategies for obtaining opioids. Notably, those in the health profession admitted to unethical practices, including prescribing opioids intended for medical procedures for personal use, encouraging patients to purchase more expensive opioids unnecessarily, and substituting prescribed opioids with cheaper alternatives for personal gain. One participant explained:

“It is because of the patients. We use this medication during labor for free, and it's available to them. If we want, we can order it from the pharmacy for them and take it for ourselves. That's how I obtain it. I used to spend 2000 birrs per day, but if I was at work, I'd prescribe it for the patients, and after they bought it, I would take it for myself and give them the substitute (tramadol). There are methods: one is to order patients to buy it, and another is to ask them to buy even from private pharmacies, taking half for myself.” [Male, 30 years old, health professional]

Another healthcare professional reported writing personal prescriptions at the workplace, i.e. One participant explained as follow:

“I bought it from a pharmacy anywhere; it was sold with a regular prescription until recently. It wasn't difficult because I wrote the prescription myself, so you could get it anywhere.” [Female, 34 years old, health professional]

Other participants reported obtaining opioids from illegal drug dealers or pharmacists, often through personal relationships, i.e. One participant shared:

“Before, it was available at the pharmacy without a prescription. After that, I found a very friendly pharmacist. I've known him for a long time, so he knows why I use it and how I will suffer if I can't get it. Otherwise, I never bought from the black market.” [Male, 22 years old, student]

### Perceived consequences of problematic opioid use

**Physical adverse consequences**: Injectable opioid users expressed concerns about injection sites, which were corroborated by visible marks and scars observed by the data collector during interviews. Many participants reported not sharing syringes and stated that they had tested negative for HIV, syphilis, and hepatitis. However, some admitted to reusing syringes.

“I don't reuse syringes. I spend a lot on pethidine, and the syringe costs only 5 Birr, so I don't see a reason not to spend on it.” [Male, 34 years old, health professional]

“I didn't share syringes and only used them once… Yes, I was tested for HIV and hepatitis, and I'm negative.” [Male, 30 years old, health professional]

Participants who engaged in binge opioid use described severe physical harm, including overdose, loss of consciousness, and injuries. Frequent fainting episodes resulted in bleeding, broken or chipped teeth, and facial and head scars.

“I took [tramadol, codeine, morphine] one time, and blood started coming from my mouth. I was unconscious for several hours; I don't know exactly how long—people told me. Usually, I woke up in about 6 minutes, but that day it took around 20 minutes, and I didn't take it again after that.” [20 years old, freshman college student]

A participant who used pethidine reported:

“This scar on my face is from an overdose. I took more than ten ampules; I don't remember when I was taken to the hospital. I lost consciousness and fell hard. When I woke up, my nose was broken, and my tooth was chipped.” [Male, 30 years old, health professional]

Participants also reported decreased appetite, gastric problems, tremors, and jerky movements. Some indicated that morphine caused more severe symptoms.

“When I was using it excessively, my short-term memory started to decline. I also developed gastric problems, tremors, and jerky movements.” [Male, 30 years old, health professional]

Others described intense physical discomfort upon cessation, attributing this to reduced endogenous opioid production.

**Psychological adverse consequences**: Participants reported depression, boredom, loneliness, anxiety, irritability, poor concentration, and reduced self-confidence. Some also experienced confusion and loss of consciousness.

“Yes, I can't walk a certain way; I think I'm going to fall, and most of the time, I'm talking to myself, walking around, saying I'm not going to fall, and worrying about what would happen if I do.” [Male, 20 years old, college student]

**Social impacts**: use led to social disconnection, reduced participation in social events, and strained relationships. Some participants, however, reported improved communication while under the influence.

“When I use pethidine, I feel like I don't need anyone. I feel complete, like I have everything and can do anything.” [Male, 34 years old, health professional]

Others described irritability and withdrawal when not using opioids.

“I separated from my lifelong friends. My character has changed over time. For example, if I take opioids today, I'll interact and talk to them peacefully, but if I don't, I become a different person.” [Male, 22 years old, student]

“Yes, you wouldn't want to talk to anyone, and it makes you irritable when the effect starts to wear off.” [Male, 20 years old, college student]

Stigma, discrimination, and relationship conflicts were also common.

“When your behaviour changes at different times of the day, your family uses you as a joke.” [Male, 20 years old, college student]

“I used to fight with my girlfriend over it.” [Male, 30 years old]

**Functional impairment**: Opioid use negatively affected education and employment, leading to absenteeism, job loss, and academic disruption.

“My education was affected. I stopped studying for two years to go to rehabilitation.” [Male, 30 years old, health professional]

“I dropped out of school because I frequently fainted.” [Male, 20 years old, college student]

**Financial impacts**: Opioid use imposed severe financial strain. Some participants resorted to drug dealing, borrowing, deception, or selling personal belongings.

“My salary was 12,500 birr (ETB)… I spent about 30,000 ETB monthly.” [Male, 28 years old, health professional]

“I could spend up to 1,500 ETB daily.” [Male, 34 years old, health professional]

Participants also reported inflated pharmacy prices and prioritization of opioids over basic needs.

**Help-seeking behaviours**: Although many participants wanted to quit opioids, barriers included financial constraints, stigma, limited information, and inadequate treatment support.

“The opioid treatment lasts up to six months but is followed by ongoing OPD follow-up… I relapsed soon after discharge.” (30-year-old health professional). Conversely, hospital-based detoxification and physician support were viewed positively.

## Discussion

This study elucidates the patterns and lived experiences of non-prescription opioid use in Ethiopia, with particular emphasis on healthcare professionals. It addresses four key objectives: 1) patterns of opioid use, 2) motivations for nonprescription use, 3) psychosocial impacts, and 4) treatment-seeking behaviours.

Consistent with previous studies (24–26), participants frequently combined opioids with alcohol and benzodiazepines. Notably, emerging combinations such as codeine with cannabis were also reported. Tramadol was the most commonly used opioid, followed by pethidine. Participants expressed a preference for consuming opioids in quiet and private settings, which were perceived to enhance euphoric effects. The observed patterns of non-medical opioid use are comparable to international experiences (3).

Motivations for opioid use were categorized into two themes: initiation and sustained use. Initiation was attributed to factors such as medical prescriptions, curiosity, peer pressure, and recreational experimentation, whereas sustained use was driven by perceived benefits. More than half of the participants reported prior use of other psychoactive substances, supporting the “gateway” theory (27) and aligning with findings from other studies (28–30). Social influences, including peer pressure and exposure through social media, also played a role, with some participants citing role models such as musicians.

Fear of withdrawal symptoms emerged as a major factor sustaining opioid use, corroborating findings from earlier research (31–33). In addition, personal adversities—consistent with the self-medication model of addiction (27)—such as divorce, unemployment, and bereavement, were linked to opioid use as a means of coping with psychological distress, including depression and somatic pain.

Despite awareness of the risks, participants developed various strategies to access opioids, including exploiting professional relationships with pharmacists. The study also documented the use of injectable opioids, such as tramadol, pethidine, and morphine. However, unlike earlier studies on heroin use in Ethiopia (12, 13), no associated infectious diseases were identified.

Experiences of opioid overdose were reported, providing critical insight into emerging and concerning trends in non-medical opioid use. Regarding treatment-seeking behaviours, stigma and fear of judgment were identified as major barriers to accessing care, particularly following relapse. Withdrawal symptoms further discouraged attempts to discontinue use.

A key strength of this study is its focus on an underexplored public health issue in Ethiopia, particularly among healthcare professionals. Methodological rigor was enhanced through triangulation and member checking. The inclusion of participants from rehabilitation centres allowed for reflective feedback on data interpretation and organization. Nevertheless, the study has limitations, including a non-representative, small, and non-random sample, as well as reliance on secondary data. These factors may restrict the validity and transferability of the findings, particularly for opioid users who do not seek healthcare services. Further research across diverse populations and settings is therefore warranted.

In conclusion, the study identified multiple high-risk patterns of opioid use, including mixing opioids with other substances, binge use, and polysubstance use. Despite restrictions on over-the-counter opioid sales in Ethiopia, participants obtained opioids through various channels, including cultivating relationships with pharmacists, using intermediaries, self-prescribing among healthcare providers, and deceptive practices.

Barriers to treatment-seeking included limited awareness of rehabilitation centres, uncertainty regarding available treatment options, concerns about the effectiveness of detoxification medications such as tramadol, and pervasive stigma. Large-scale, community-based studies in both urban and rural settings are needed to better estimate the burden of opioid use. Additionally, intervention research aimed at improving access to care and developing harm-reduction guidelines is essential.

## Data Availability

The data supporting the findings of this study are available from the corresponding author, M.T., upon reasonable request.
